# In Vitro and In Vivo Nutraceutical Characterization of Two Chickpea Accessions: Differential Effects on Hepatic Lipid Over-Accumulation

**DOI:** 10.3390/antiox9030268

**Published:** 2020-03-24

**Authors:** Mariangela Centrone, Patrizia Gena, Marianna Ranieri, Annarita Di Mise, Mariagrazia D’Agostino, Maria Mastrodonato, Maria Venneri, Davide De Angelis, Stefano Pavan, Antonella Pasqualone, Carmine Summo, Valentina Fanelli, Giovanna Valenti, Giuseppe Calamita, Grazia Tamma

**Affiliations:** 1 Department of Biosciences Biotechnologies and Biopharmaceutics, University of Bari Aldo Moro, 70125 Bari, Italy; mariangela.centrone@uniba.it (M.C.); annapatrizia.gena@uniba.it (P.G.); marianna.ranieri@uniba.it (M.R.); annarita.dimise@uniba.it (A.D.M.); mariagrazia.dagostino@uniba.it (M.D.); maria.venneri@uniba.it (M.V.); giovanna.valenti@uniba.it (G.V.); giuseppe.calamita@uniba.it (G.C.); 2 Department of Biology, University of Bari Aldo Moro, 70125 Bari, Italy; maria.mastrodonato@uniba.it; 3 Department of Soil, Plant and Food Sciences, University of Bari Aldo Moro, 70125 Bari, Italy; davide.deangelis@uniba.it (D.D.A.); stefano.pavan@uniba.it (S.P.); antonella.pasqualone@uniba.it (A.P.); carmine.summo@uniba.it (C.S.); valentina.fanelli@uniba.it (V.F.); 4 Istituto Nazionale di Biostrutture e Biosistemi, (I.N.B.B.), 00136 Rome, Italy

**Keywords:** legumes, liver, hepatic steatosis, lipid dyshomeostasis, ROS

## Abstract

Dietary habits are crucially important to prevent the development of lifestyle-associated diseases. Diets supplemented with chickpeas have numerous benefits and are known to improve body fat composition. The present study was undertaken to characterize two genetically and phenotypically distinct accessions, MG_13 and PI358934, selected from a global chickpea collection. Rat hepatoma FaO cells treated with a mixture of free fatty acids (FFAs) (O/P) were used as an in vitro model of hepatic steatosis. In parallel, a high-fat diet (HFD) animal model was also established. In vitro and in vivo studies revealed that both chickpea accessions showed a significant antioxidant ability. However, only MG_13 reduced the lipid over-accumulation in steatotic FaO cells and in the liver of HFD fed mice. Moreover, mice fed with HFD + MG_13 displayed a lower level of glycemia and aspartate aminotransferase (AST) than HFD mice. Interestingly, exposure to MG_13 prevented the phosphorylation of the inflammatory nuclear factor kappa beta (NF-kB) which is upregulated during HFD and known to be linked to obesity. To conclude, the comparison of the two distinct chickpea accessions revealed a beneficial effect only for the MG_13. These findings highlight the importance of studies addressing the functional characterization of chickpea biodiversity and nutraceutical properties.

## 1. Introduction

Lifestyle and diet patterns play a critical role in disease development. Several studies demonstrate that healthy aging is often associated with a plant-based diet [1]. In contrast, the so-called Western diet, which is enriched in red meat and fats, combined with sedentary a lifestyle, significantly increases the risk of developing metabolic and energy balance disorders including insulin resistance, hyperlipidemia, hepatic steatosis, and obesity, that are hallmarks of the metabolic syndrome [2]. Nowadays, unhealthy lifestyle and diet-associated diseases can be considered a worldwide rising issue. Therefore, there is a growing interest in characterizing selective food groups for their physiological benefits, which could be useful to prevent chronic disease risk [3].

Legumes are a fundamental component of the diet of several human populations and chickpea (*Cicer arietinum* L.) represents the second most widely cultivated grain legume worldwide [4]. Chickpeas are characterized by having a high content of carbohydrates, proteins, fats, vitamins, and fibers [5] that reduce the risk of incidence of chronic diseases [6]. Numerous studies have demonstrated that chickpeas and ethanol extract of chickpeas can alter lipid metabolism by reducing the formation of lipid droplets during 3T3-L1 adipocytes differentiation [7]. Moreover, the consumption of chickpeas slows down the glycemic response [8] and weakens insulin resistance, type-2 diabetes, and dyslipidemia associated with a high-fat diet (HFD) [9].

A HFD is associated with the overproduction of reactive oxygen species (ROS). Imbalances between oxidants and antioxidant endogenous reserve play a role in the onset of several disorders [10]. Indeed, a HFD increases the expression level of nicotinamide adenine dinucleotide phosphate (NADPH) oxidase while reduces antioxidant enzymes [11,12]. Cellular ROS enhance lipid peroxidation, thereby possibly altering the expression and the activity of the oxidative-sensitive nuclear factor kappa beta (NF-kB) and other chemokines [12]. NF-kB is a crucial player in controlling various cellular processes such as inflammation, cell survival, and proliferation. NF-kB is normally sequestered in the cytosol by binding the inhibitory proteins named IkBs [13]. Two signaling pathways can lead to NF-kB activation [14]. The main mechanism for the classical stimulation of the NF-kB pathway includes phosphorylation of IkB by the IκB kinase (IKK), its ubiquitylation and degradation. These molecular events promote the activation and nuclear translocation of NF-kB [14]. Phosphorylation of NF-kB at serine-536, by multiple kinases, provides an additional level of regulation of NF-kB that affects cell proliferation by controlling the expression of numerous cell cycle regulatory proteins [15,16] such as cyclin D1 and proliferating cell nuclear antigen (PCNA) [17]. The canonical pathway is switched on by various cytokines, such as TNFα that increases significantly secondary to a HFD [18]. Numerous studies have strongly indicated that food habits play a key role in the development of several disorders. Medical plants and a balanced diet have been reported to slow down chronic diseases. However, evidence for the consumption of chickpeas in dyslipidemia, insulin resistance, and type 2 diabetes are few and sometimes discordant [19,20].

Recent studies on global chickpea germplasm collections reveal a high level of genetic, phenotypic, and compositional diversity [4,21,22]. The present study was carried out to investigate the physiological effect of two genetically and phenotypically diverse chickpea accessions in a cellular and animal model of nonalcoholic fatty liver disease.

## 2. Materials and Methods

### 2.1. Chemicals and Antibodies

All chemicals were obtained from Merck (Merck KGaA, Darmstadt, Germany). Monoclonal antibodies against pNF-kB p65 (27.Ser 536), NF-kB p65 (F-6), and PCNA (PC10) were purchased from Santa Cruz Biotechnology (Tebu Bio, Milan, Italy). Secondary anti-mouse conjugated to horseradish peroxidase (HRP) was obtained from Bio-Rad (Bio-Rad Laboratories, Inc., Hercules, CA, USA). The tert-butyl hydroperoxide (tBHP) was a kind gift from A. Signorile (University of Bari, Aldo Moro, Bari. Italy). The RayBio^®^ Mouse Insulin ELISA Kit was from RayBiotech (Norcross, GA, USA).

### 2.2. Chickpea Selection and Preparation of Extracts

Two chickpea accessions, namely MG_13 and PI358934, were selected from the germplasm collections previously characterized, as they belong to distinct genetic clusters and display markedly different phenotypic features [22,23]. For each accession, 40 plants were grown at the experimental farm “P. Martucci” of the University of Bari (41°01′22.1″ N and 16°54′21.0″ E) from 2013 to 2014. At crop maturity, seeds were bulked separately for each accession and were cleaned to remove broken seeds, dust, and other undesirable matter. To obtain the wholemeal flour with uniform particle size, the seeds were ground by an electric mill (Model ETA, Vercella Giuseppe, Mercenasco, Italy) equipped with a sieve of 0.6 mm. The chemical and nutritional composition of the two chickpea accessions is reported in the Supplementary Material of a study by Summo et al. [4]. MG_13 is characterized by higher protein and lower lipid content than PI358934, as well as by higher oleic acid and lower linoleic acid content. For the preparation of the extracts, 2 g of chickpea flour were mixed with 20 mL of a 30/70 (*v/v*) ethanol/water solution in centrifuge tubes and stirred for 2 h in the dark. Then, the extracts were centrifuged at 12,000× *g* for 10 min. The supernatant was recovered and filtered through nylon filters (pore size 0.45 μm). Finally, the extracts were freeze-dried by a laboratory lyophilizer (Analytical Control De Mori s.r.l., Milano, Italy) and stored at −20 °C until the analysis.

### 2.3. Cell Culture, Treatments, Crystal Violet Assay, and ROS Detection

Rat hepatoma FaO cells were grown in Coon′s modified Ham′s F12 (Euroclone, Milan, Italy) supplemented with 10% fetal bovine serum (FBS) (Thermo Fisher Scientific, Waltham, MA, USA), 100 I. u./mL penicillin, 100 µg/mL streptomycin (Euroclone, Milan, Italy) at 37 °C in 5% CO_2_ and used at 70% to 80% confluence. For treatments, cells were incubated for 24 h in high glucose medium, without FBS, supplemented with 0.25% bovine serum albumin (BSA) and exposed to chickpea extracts obtained from the accessions MG_13 and PI358934, respectively. Cells were left under basal condition or stimulated with a mixture of free fatty acids (FFAs) (0.5 mM oleate and 0.25 mM palmitate) for 3 h [24,25].

Crystal violet assay was performed as previously described [26]. Cells viability was detected by measurement of the optical density at 595 nm (DO595) with a microplate reader (Bio-Rad Laboratories, Inc., Hercules, CA, USA).

ROS were detected as previously shown [26,27] and using the dihydrorhodamine-123 (10 μM). The fluorescence emission signal was recorded using a fluorimeter (RF-5301PC, Shimadzu Corporation, Kyoto, Japan) at excitation and emission wavelengths of 508 and 529 nm, respectively.

### 2.4. Lipid Inclusions Staining

FaO cells were fixed with 4% paraformaldehyde (PFA) in phosphate buffer saline (PBS) for 20 min. After washing in 50% isopropanol, cells were stained with 0.12% Oil Red O solution and counterstained with hematoxylin and eosin. Alternatively, livers were fixed quickly in 4% PFA, dehydrated through a graded ethanol series and embedded in paraffin wax. Lipid inclusions analysis was performed as previously described [26,28]. In paraffin sections stained with periodic acid-Schiff (PAS), lipid droplets were characterized by circular shape and chromatic uniformity (white areas, because the lipids have been removed from the droplets by the solvent used for dehydration). The obtained data were analyzed using an Image J–Particles Analyzer (https://imagej.net). Statistical analysis was performed with GraphPad Prism (GraphPad Software, San Diego, CA, USA) by one-way ANOVA followed by Dunnett’s Multiple Comparisons test with * *p* < 0.05 considered statistically different.

### 2.5. Cell and Liver Lysates

FaO cells were seeded onto 60 mm diameter Petri dishes. After treatments, cells were lysed as previously described [29]. Alternatively, 30 to 50 mg of frozen liver tissue per mouse sample was homogenized with a mini-potter in an ice-cold RIPA buffer, in the presence of proteases (1 mM PMSF, 2 mg/mL leupeptin, and 2 mg/mL pepstatin A) and phosphatases (10 mM NaF and 1 mM sodium orthovanadate) inhibitors. Suspensions were then centrifuged at 12,000× *g* for 10 min at 4 °C. Lysates were used for SDS-PAGE and Western blotting analysis.

### 2.6. Gel Electrophoresis and Western Blotting

Proteins were separated using 10% or 12% stain-free polyacrylamide gels (Bio-Rad Laboratories, Inc., Hercules, CA, USA) under reducing conditions as previously described [26]. Protein bands were electrophoretically transferred onto Immobilon-P membranes (Merck KGaA, Darmstadt, Germany) for Western blot analysis, blocked in TBS-Tween-20 containing 3% bovine serum albumin (BSA) and incubated with primary antibodies overnight. Immunoreactive bands were detected with secondary goat anti-mouse horseradish peroxidase-coupled antibodies. Membranes were incubated with Super Signal West Pico Chemiluminescent Substrates (Thermo Fisher Scientific, Waltham, MA, USA), and the signals were visualized with the ChemiDoc System gels (Bio-Rad Laboratories, Inc., Hercules, CA, USA). Obtained bands were normalized to total protein using the stain-free technology gels (Bio-Rad Laboratories, Inc., Hercules, CA, USA). Densitometry analysis was performed using Image Lab gels (Bio-Rad Laboratories, Inc., Hercules, CA, USA). Data were analyzed using GraphPad Prism (GraphPad Software, San Diego, CA, USA).

### 2.7. Animals and Experimental Protocol

Forty-five C57BL/6J male mice (Envigo RMS S.R.L., San Pietro al Natisone, Udine, Italy), 3 weeks old and about 18 g of initial body weight were housed in a temperature-controlled room (25 °C) on a 12 h light-dark cycle. Mice were fed with a control diet (CTR) for one week. Mice had free access to water and diet according to the assigned dietary formulation for 16 weeks. The four kinds of diets (Altromin Rieper SpA, Vandoies, Italy) were the following: (1) a standard control diet (CTR) containing 10% fat, 24% proteins, and 66% of carbohydrates (metabolized energy 3.514 kcal/kg diet); (2) a high-fat diet (HFD) made of 45% fat, 19% proteins, and 36% of carbohydrates (metabolized energy 4.496 kcal/kg diet) (3) a high-fat plus chickpea diet (accession number: MG_13) (HFD + MG_13) having the same metabolized energy of the HFD diet, as well as the same composition except that 10% of MG_13 raw crushed chickpea seed flour replaced crude fibers and ashes of HFD; (4) a high-fat plus chickpea diet (accession number: PI358934) (HFD + PI358934) similar to the previous one except that 10% of PI358934 raw crushed chickpea seed flour replaced crude fibers and ashes of HFD. The experimental diets were stored at −20 °C to avoid rancidity.

Before killing by cervical dislocation, mice were deprived of food for 12 h. Blood glucose levels were measured from the tail vein using a freestyle glucometer (Accu-Check Aviva, Roche, Basel, Switzerland). Livers were removed quickly, weighed, and fixed for histochemical analysis or frozen using liquid N_2_. The serum was separated from blood samples and promptly stored at −80 °C.

Animal experiments were carried out following the Directive 2010/63/UE, enforced by Italian D.L. 26/2014, and approved by the animal care and the Committee of the University of Bari (OPBA), Bari, Italy and the Italian Ministry of Health, Rome, Italy (authorization n.326/2018-PR).

### 2.8. Serum Insulin Measurement

Serum insulin secretion was determined using an ELISA kit, following the manufacturer’s instructions. Briefly, each sample was incubated for 2.5 h at room temperature with gentle shaking. In parallel, increasing concentrations (400, 200, 100, 50, 25, 12.5, and 6.25 μlU/mL) of a standard protein reproducing mouse insulin were incubated as an internal standard. Wells were washed with the appropriate washing solution and incubated with biotinylated antibodies for 1 h at room temperature. Then, wells were washed and incubated with streptavidin solution for 45 min at room temperature. After the last four washes, TMB substrate reagent was added to each well and incubated for 30 min in the dark at room temperature. Optical density was measured at 450 nm by a microplate reader (iMark, Bio-Rad Laboratories, Inc., Hercules, CA, USA).

### 2.9. Statistical Analysis

All values are reported as means ± S.D. Statistical analysis was performed by one-way ANOVA followed by Dunnett’s multiple comparisons test with * *p* < 0.05 considered statistically different.

## 3. Results

### 3.1. Characterization of the Chickpeas Extracts in FaO Cells

**Figure 1:**
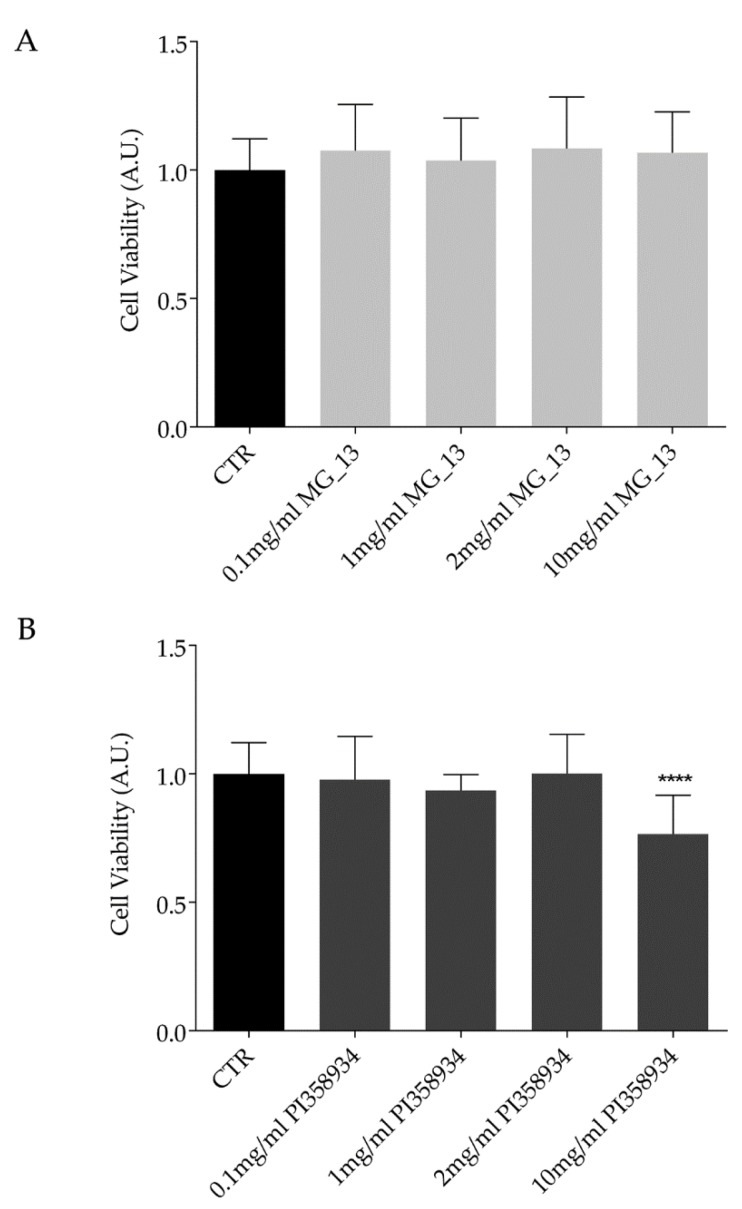
Cell viability of FaO cells exposed to the chickpea extracts. FaO cells were left under basal condition (CTR) or exposed to different concentrations (0.1 mg/mL, 1 mg/mL, 2 mg/mL, or 10 mg/mL) of chickpea extract MG_13. (**A**) and PI358934; (**B**) as described in the Methods Section. Cells were stained with crystal violet solution. Data are presented as means ± S.D. of 3 independent experiments and analyzed by one-way ANOVA followed by Dunnett’s multiple comparisons test. (**** *p* < 0.0001 vs. control diet (CTR)).

**Figure 2:**
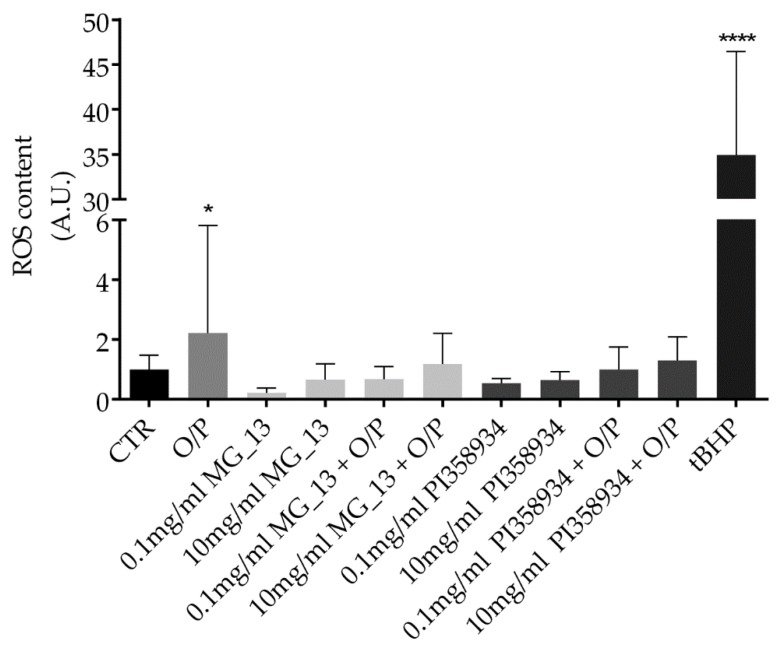
Reactive oxygen species (ROS) content in FaO cells. ROS content was measured using dihydrorhodamine-123 fluorescence in FaO cells treated as described in the Methods Section. As a positive control, cells were treated with tert-butyl hydroperoxide (tBHP). Data are shown as means ± S.D. of 3 independent experiments and analyzed by one-way ANOVA followed by Dunnett’s multiple comparisons test. (* *p* < 0.05 vs. CTR and **** *p* < 0.0001 vs. CTR).

Chickpeas have been repeatedly reported to have a beneficial effect in preventing chronic diseases and reducing adiposity. However, a clear demonstration of the health benefits of chickpeas is still missing. In this study, chickpea extracts obtained from two different accessions (MG_13 and PI358934) of a global chickpea collection [4,22] have been characterized. The potential effects of the extracts on cell viability were evaluated by performing the crystal violet assay. FaO cells were exposed for 24 h to increasing concentrations of the chickpea extracts derived from the two accessions (0.1 mg/mL, 1 mg/mL, 2 mg/mL, and 10 mg/mL). Compared to the untreated condition (CTR), treatment with the extract isolated from the accession MG_13 did not alter cell viability at the concentrations used in this study (Figure 1A). In contrast, treatment with the extract obtained from the accession PI358934 reduced cell viability at the highest concentration (10 mg/mL) (**** *p* < 0.0001 vs. CTR). No relevant cytotoxic effect was observed at 0.1 mg/mL, 1 mg/mL, and 2 mg/mL doses (Figure 1B). As with other pulses, chickpea seeds contain several bioactive compounds including anthocyanins, carotenoids, and phenols [4] which are important to counteract oxidative stress. Reactive species can potentially promote inflammation and fibrosis during the development of hepatic steatosis. Thus, to investigate the functional effects of chickpea extracts and their correlation with oxidative signals and inflammation, a liver steatosis cellular model was established by exposing FaO cells to 0.75 mmol/L oleate/palmitate (O/P) for 3 h [24,25,30]. Compared to the untreated cells (CTR), exposure to O/P led to a significant increase of the reactive oxygen species (ROS) as assessed by a fluorimetric method (* *p* < 0.05 vs. CTR). As an internal and additional positive control, cells were treated with the oxidant tert-butyl hydroperoxide showing a relevant increase of ROS content as compared with that measured in CTR cells (**** *p* < 0.0001 vs. CTR). Exposure to both chickpea extracts at 0.1 mg/mL and 10 mg/mL displayed a similar ability to decrease intracellular ROS induced by the O/P treatment (Figure 2). Hence, the lowest (0.1 mg/mL) concentration of the extracts was used in the following experiments.

### 3.2. Effects of Chickpea Extracts on Lipid Accumulation in FaO Cells

**Figure 3:**
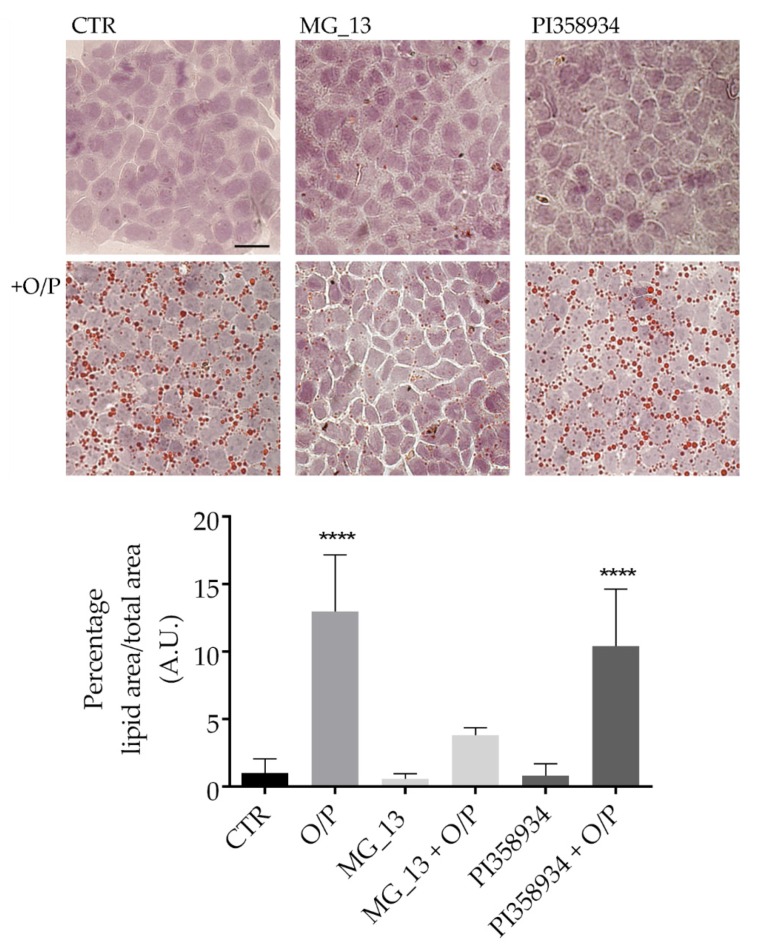
Detection of intracellular lipid droplets. FaO cells were left under basal condition (CTR) or treated with MG_13 (0.1 mg/mL), PI358934 (0.1 mg/mL) or with chickpea extract in the presence of a mixture of free fatty acids (FFAs) (oleate/palmitate). Oil Red O staining revealed lipid droplet formation. Lipid droplets are stained dark red and were analyzed using Image-J software (Bar 25 µm). Data are shown as a percentage of lipid area/total area and are presented as means ± S.D. of 3 independent experiments and analyzed by one-way ANOVA followed by Dunnett’s multiple comparisons test. (**** *p* < 0.0001 vs. CTR).

Lipid accumulation was visualized using Oil Red O staining. This staining can detect neutral triglycerides and glycolipids in the control (CTR) and steatotic hepatocytes (O/P) exposed or not to the chickpea extracts (0.1 mg/mL) for 24 h (Figure 3). Treatment with O/P promoted lipid droplet over-accumulation that was not observed in the cells pretreated with the extract obtained from the MG_13 accession (MG_13 + O/P). In contrast, exposure to the extract isolated from the PI358934 accession did not hamper the over-accumulation of fat induced by the O/P mixture (PI358934 + O/P). Compared to the untreated cells, the O/P treatment resulted in a significant increase in lipid accumulation (**** *p* < 0.0001 vs. CTR). Similar to CTR cells, incubation with each one of the extracts from the two accessions did not lead to an over-accumulation of lipids within the cells.

### 3.3. Effects of Chickpea Extracts on NF-kB Phosphorylation in FaO Cells

**Figure 4:**
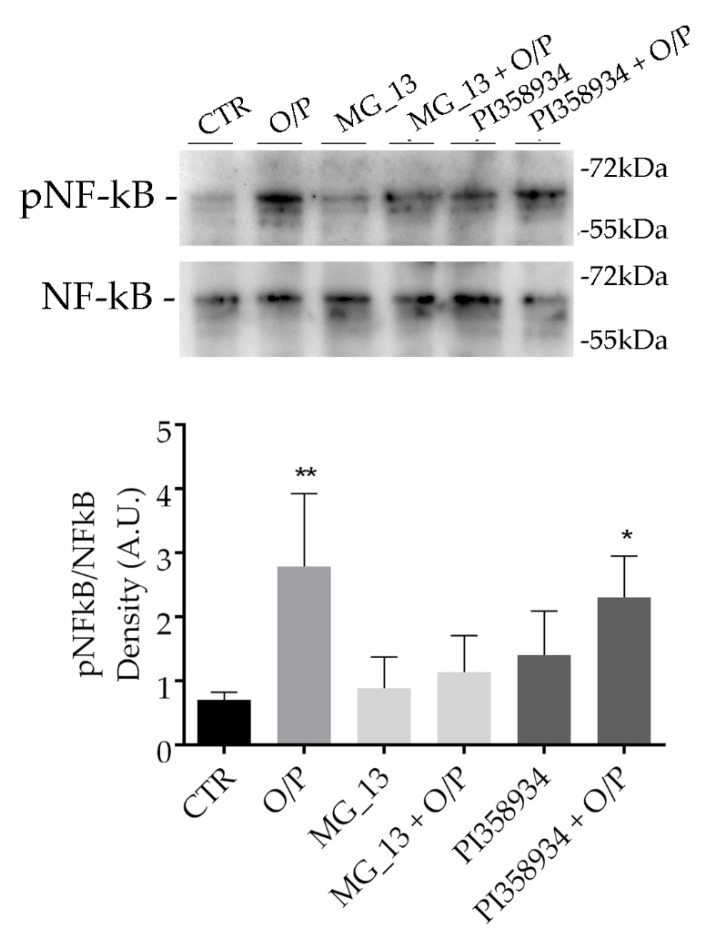
Effects of the chickpea extracts on nuclear factor kappa beta (NF-kB) phosphorylation. FaO cells were treated as described in the Methods Section. An equal amount of proteins (30 μg/lane) was separated by gel electrophoresis and immunoblotted for evaluation of pNF-kB and total NF-kB levels. Densitometric analysis revealed that the O/P treatment caused a significant increase in pNF-kB levels as compared with cells under basal condition (CTR). Treatment with MG_13 (0.1 mg/mL) prevented the O/P-dependent increase of pNF-kB. In contrast, treatment with PI358934 (0.1 mg/mL) did not impair the O/P-action. No significant alterations in pNF-kB levels were observed by the sole treatment with chickpea extract. Data are expressed as means ± S.D. and were analyzed by one-way ANOVA followed by Dunnett’s multiple comparisons test. (** *p* < 0.01 vs. CTR and * *p* < 0.05 vs. CTR, *n* = 4).

Western blotting analysis revealed that in steatotic hepatocytes (O/P), phosphorylation of NF-kB at serine-536 significantly increased as compared with untreated cells (** *p* < 0.01 vs. CTR) (Figure 4). Preincubation with the extract obtained from the MG_13 accession impaired the increase of NF-kB phosphorylation at S536 induced by O/P (MG_13 + O/P). In contrast, phosphorylation of NF-kB significantly increased (* *p* < 0.05 vs. CTR) in steatotic hepatocytes pretreated with the extract PI358934 (PI358934 + O/P) at a similar level to that observed in O/P treated cells. Treatment with only the extracts, in the absence of O/P, did not alter NF-kB phosphorylation (Figure 4).

### 3.4. Animal Studies: Body Weight, Food Intake, Food Efficiency Ratio

To further investigate the biological actions of the two chickpea accessions, an animal model of nonalcoholic fatty liver disease (NAFLD) was established by feeding mice with a high-fat diet. As already described in the Methods Section, four different diets were randomly assigned to C57BL/6J adult male mice for 16 weeks. The initial and final body weights, the body weight gain, the food intake, and the feed efficiency ratios (FER) of the mice are indicated in Table 1. Compared to CTR mice, the final body weight and weight gain were significantly higher in the HFD groups (**** *p* < 0.0001). The addition of the flour obtained from the accession PI358934 (HFD + PI358934) significantly increased the final body weight and the FER with respect to those measured in HFD mice (^####^ *p* < 0.0001).

### 3.5. Serum Parameters and Liver Function

Selected serum parameters are listed in Table 2 showing that glycemia was higher in the HFD groups as compared with that measured in the control mice. However, the serum glucose measured in the HFD + MG_13 group was significantly lower than the glycemia measured in the HFD mice (^#^ *p* < 0.05 vs. HFD). Serum insulin levels were slightly changed among groups although these differences were not statistically significant (*p* > 0.05). Total cholesterol was increased in mice fed with HFD in the presence of chickpea flours more than in the group kept on the HFD diet (HFD + MG_13 vs. CTR, ** *p* < 0.01 and HFD + PI358934 vs. CTR, ** *p* < 0.01). However, the increase measured in the HFD + PI358934 mice was higher than the one measured in the HFD mice (HFD + PI358934 vs. HFD, ^#^ *p* < 0.05). Serum triglycerides were increased in the HFD mice that received the flours. To evaluate the liver function, the levels of alkaline phosphatases and the aspartate aminotransferase (AST) were determined. Interestingly, the alkaline phosphatases level did not change among groups. In contrast, a significant increase of the AST was found in the HFD and HFD + PI358934 groups as compared with the control mice (HFD vs. CTR, * *p* < 0.05 and HFD + PI358934 vs. CTR, ** *p* < 0.01). No significant alteration was measured in the HFD + MG_13 animals as compared with the CTR and HFD groups. Moreover, the liver weight normalized to the total body weight significantly increased only in mice fed with HFD + PI358934 (HFD + PI358934, 0.0394 ± 0.0031 vs. CTR: 0.0303 ± 0.0005, ** *p* < 0.01).

### 3.6. Effects of Chickpea Diets on Lipid Accumulation and NF-kB Phosphorylation in Liver

**Figure 5:**
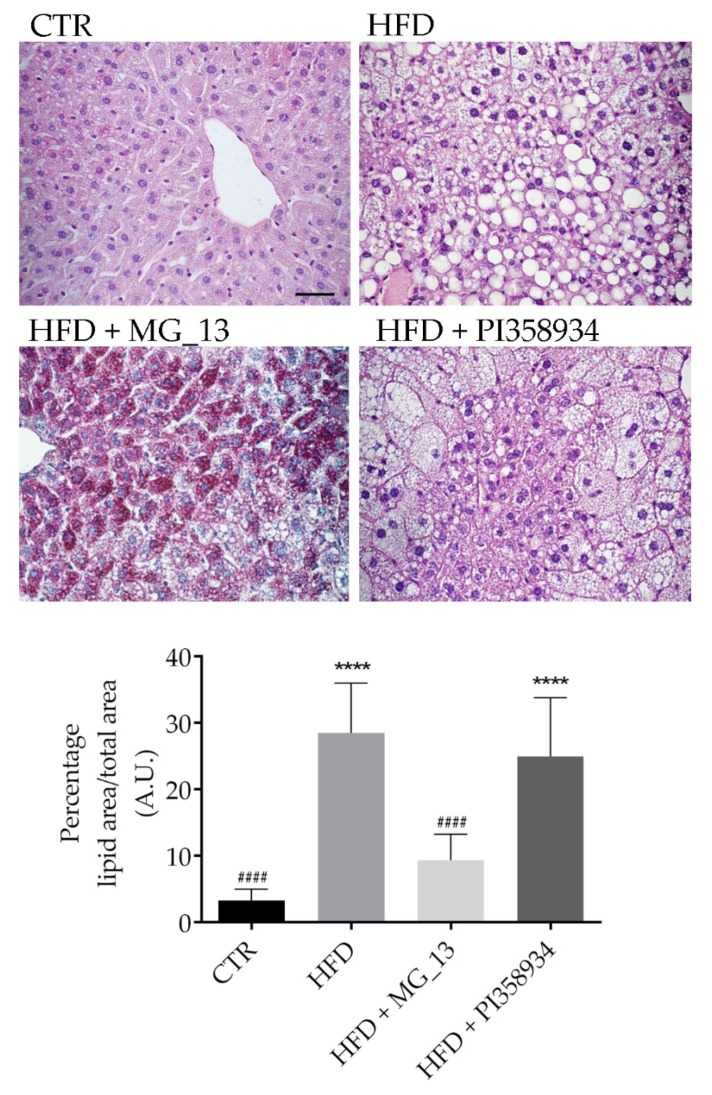
Histochemical features of liver parenchyma of mice. Paraffin sections of mice livers were stained with PAS-hematoxylin and eosin. PAS reaction demonstrated glycogen stores (stained red) in the hepatocytes. The lipid droplets being approximately circular in the section were counted with Image-J software (Bar 50 µm). Data are shown as a percentage of lipid area/total area and are presented as means ± S.D. of 3 independent experiments and analyzed by one-way ANOVA followed by Dunnett’s multiple comparisons test. (**** *p* < 0.0001 vs. CTR and ^####^ *p* < 0.0001 vs. HFD).

**Figure 6:**
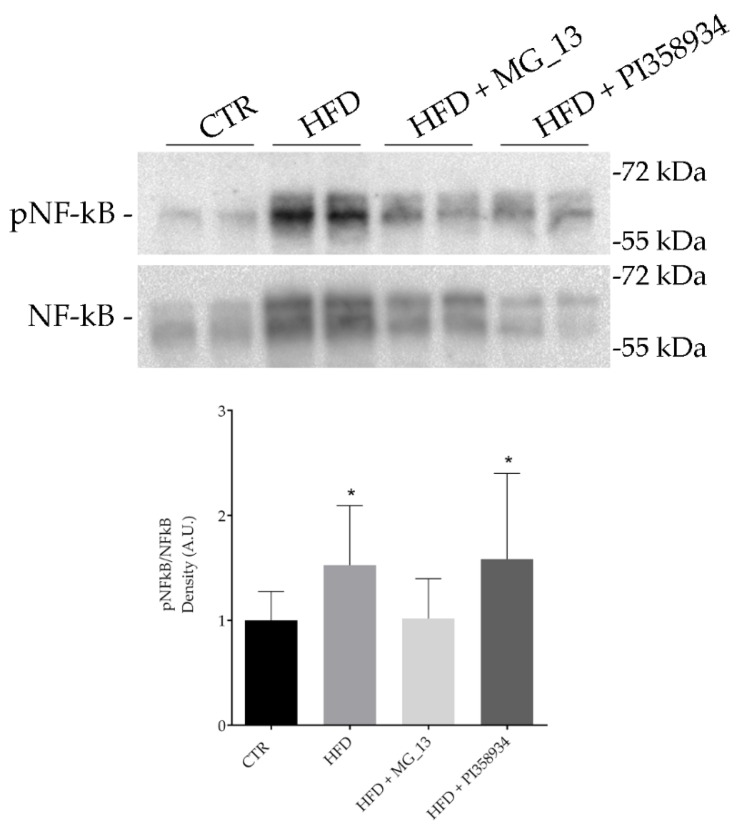
Effects of chickpea diets on NF-kB phosphorylation. Liver lysates were prepared as described in the Methods Section. An equal amount of proteins (30 μg/lane) was separated by gel electrophoresis and immunoblotted for the evaluation of pNF-kB and total NF-kB levels. Densitometric analysis revealed that the HFD diet (HFD, *n* = 12) caused a significant increase in pNF-kB levels as compared with the mice fed with a normal diet (CTR, *n* = 12). The diet with HFD + MG_13 (*n* = 12) prevented the HFD dependent increase of pNF-kB. Data are expressed as means ± S.D. and were analyzed by one-way ANOVA followed by Dunnett’s multiple comparisons test (* *p* < 0.05 vs. CTR).

Lipid inclusions analysis in the liver revealed that HFD feeding promotes the over-accumulation of lipid droplets (**** *p* < 0.0001 vs. CTR) (Figure 5). A similar increase in lipid accumulation was found in liver sections prepared from the HFD + PI358934 fed mice, likely suggesting that this accession has no beneficial effect on hepatic lipid over-accumulation (**** *p* < 0.0001 vs. CTR). In contrast, a significant reduction in lipid droplets over-accumulation as compared with the HFD samples was detected in liver sections of the HFD + MG_13 fed group (^####^ *p* < 0.0001 vs. HFD), likely indicating that this accession prevented liver adiposity. Indeed, an increase of the cytoplasmic glycogen concentration was found in the HFD + MG_13 mice as assessed by PAS staining. NF-kB can be considered a general biomarker of inflammation and is often activated in patients with dyslipidemia. The Western blotting analysis (Figure 6) of liver lysates revealed that NF-kB phosphorylation at S536 increased in the HFD and HFD + PI358934 fed mice (* *p* < 0.05 vs. CTR). In contrast, the degree of NF-kB phosphorylation in the HFD + MG_13 fed mice was similar to that obtained in the liver lysates of the CTR mice.

### 3.7. Effects of Chickpea Diets on ROS Production and Proliferating Cell Nuclear Antigen (PCNA) Expression in Liver

**Figure 7:**
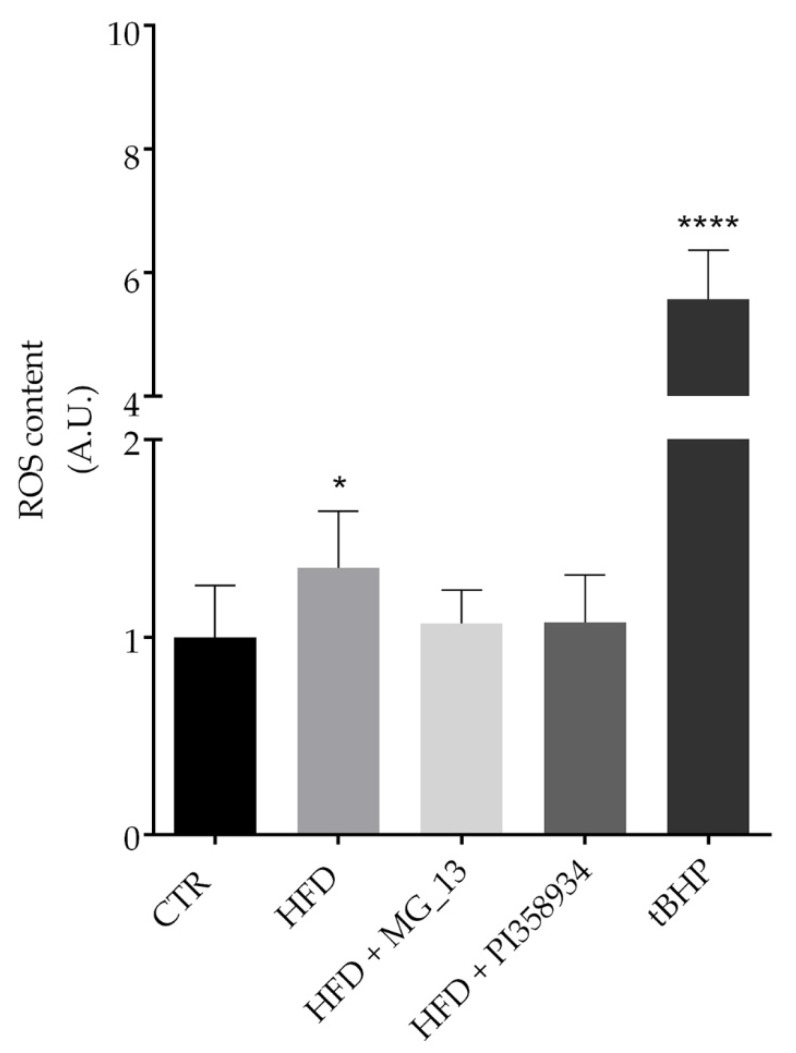
ROS content in liver tissue. ROS content was measured using dihydrorhodamine-123 fluorescence in a liver slice as described in the Methods Section. As a positive control, the liver sections were treated with tBHP. Data are expressed as means ± S.D. and analyzed by one-way ANOVA followed by Dunnett’s multiple comparisons tests (**** *p* < 0.0001 vs. CTR and * *p* < 0.05 vs. CTR).

**Figure 8:**
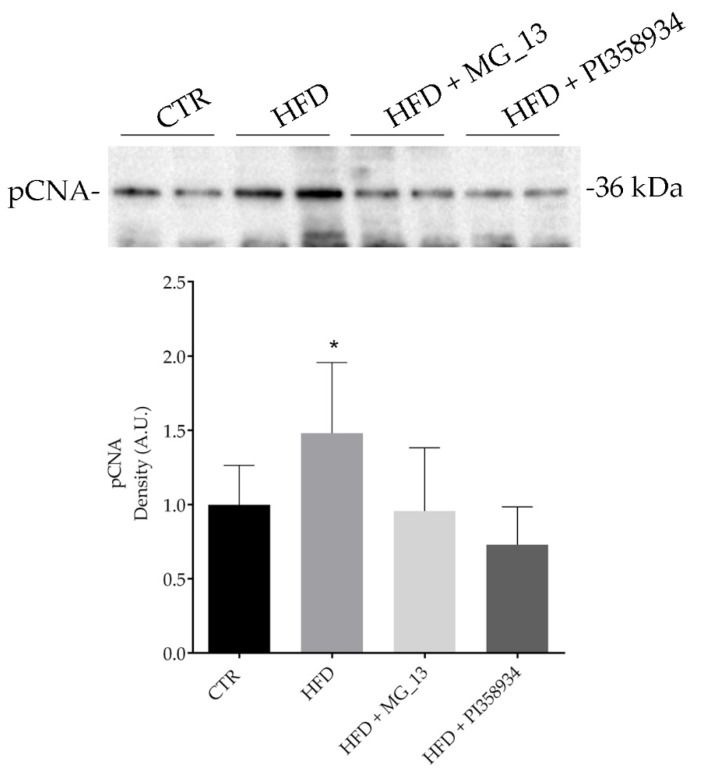
Effects of chickpea diets proliferating cell nuclear antigen (PCNA) expression. Liver lysates were prepared as described in the Methods Section. An equal amount of proteins (30 μg/lane) was separated by gel electrophoresis and immunoblotted for evaluation of the PCNA levels. Densitometric analysis revealed that the HFD diet (HFD) caused a significant increase in the PCNA levels as compared with mice fed a normal diet (CTR). No significant alterations in PCNA levels were observed in mice fed the HFD + MG_13 diet or HFD + PI358934 diet. Data are expressed as means ± S.D. and analyzed by one-way ANOVA followed by Dunnett’s multiple comparisons test (* *p* < 0.05 vs. CTR).

Fresh liver sections were subjected to a fluorimetric assay to detect ROS content as described in the Methods Section (Figure 7). Compared to the CTR group, the HFD groups displayed a significant increase in ROS content (* *p* < 0.05 vs. CTR). In contrast, a relevant decrease in ROS production was found in the livers of the HFD mice that received the chickpea flours, likely indicating that the consumption of these flours exerts an antioxidant effect. The Western blotting analysis of liver lysates (Figure 8) revealed that the expression of PCNA, a marker of cell proliferation, increased in the HFD fed mice as compared with the CTR group. Interestingly, the expression level of PCNA in liver lysates of the HFD + MG_13 and HFD + PI358934 fed mice was significantly lower as compared with lysates from the HFD fed animals, and thereby similar to that detected in lysates of CTR animals (HFD vs. CTR, * *p* < 0.05)

## 4. Discussion

Overweight and obesity associated with a high-fat diet have become a global health concern having a tremendous impact on clinical care [31]. High-fat diets increase the risk of the incidence of several clinical disorders such as dyslipidemia, hypertension, type-2 diabetes, and liver diseases [32,33]. It has been reported, that prolonged high-fat feeding promoted nonalcoholic fatty liver disease (NAFLD) that can lead to nonalcoholic steatohepatitis (NASH) and cirrhosis [34,35]. Therefore, the identification of new dietary supplements or the characterization of specific foods that reduce hepatic fat over-accumulation and improve the systemic lipid profile is an unmet need. The present study was undertaken to investigate the possible beneficial effects of dietary consumption of chickpeas. Legumes are traditionally recommended to reduce lipid accumulation and to prevent insulin resistance, possibly because they provide several nutrients including low glycemic index carbohydrates and dietary fibers [36]. However, clear evidence is missing for the recommendation of chickpeas in controlled diets with the aim of improving health conditions. It is not clear at what amount beneficial effects can be gained. Short- and long-term consumption leads to differential responses of glycemia, insulin, and other serum parameters [19]. Another crucial aspect to be investigated is whether genetic diversity and a different composition in cultivated material is an important variable for determining the effects of chickpea consumption on human health [19,20,36]. Here, both in vitro and in vivo studies were performed to compare the effects of two genetically and phenotypically diverse chickpea accessions on lipid accumulation, ROS generation, and the activation of the inflammatory factor NF-kB. Exposure of hepatic FaO cells to free fatty acids (FFAs) mimicked cellular steatosis [30]. Accordingly, in this report, Oil Red O staining revealed the presence of numerous intracellular lipid droplets in O/P treated FaO cells. Furthermore, abnormal intrahepatic fat accumulation led to a considerable upregulation of the oxidative signals in NAFLD patients [34]. Fluorimeter measurements showed that the O/P treatment promoted ROS generation as compared with the untreated cells. Interestingly, both chickpea accessions reduced the ROS production induced by the O/P treatment. The significant antioxidant effect could be related to the presence, in these chickpea accessions, of bioactive molecules such as carotenoids, anthocyanins, and phenols [23]. Oxidative stress and inflammation are involved in the onset and progression of numerous diseases. Therefore, phytocoumpounds, such as phenols with proven antioxidant ability, are considered health-promoting natural antioxidants [37,38]. Higher production of reactive species can occur secondary to intracellular altered lipid metabolism and accumulation [34]. However, only the accession MG_13 prevented lipid over-accumulation induced in steatotic hepatocytes by the O/P treatment. It is well established that HFD feeding causes an increase of intrahepatic fat, abnormal functions of lysosomes and mitochondria, leading to the activation of NF-kB [39,40]. The nuclear factor NF-kB is a master regulator of inflammatory and oxidative signals. Numerous reports have shown that metabolic diseases associated with obesity can be accompanied by abnormal NF-kB expression and activity [30,41]. In 12-weeks feeding HFD mice, a significant upregulation of NF-kB signaling was also demonstrated [41]. Therefore, to further investigate the differential effects of the two chickpea accessions, the activity of NF-kB was evaluated. Steatotic FaO cells displayed a significant increase of NF-kB phosphorylation at serine 536, consistent with an activation of the NF-kB pathway. To confirm the in vitro findings, an HFD mouse model was established. As predicted, HFD feeding increased total body weight. However, mice receiving the flour of the accession PI358934 displayed a higher body weight as compared with CTR and HFD mice. A similar trend was also found for the food efficiency ratio (FER). These observations suggested that animals fed with the PI358934 accession gained more weight as compared with the HFD + MG_13 fed mice. Moreover, compared to CTR mice, HFD feeding increased blood glucose. Interestingly, MG_13 supplementation was associated with a significant reduction of the hyperglycemia induced by HFD, likely indicating that the consumption of this accession could have a beneficial effect in decreasing blood glucose. The supplementation of the accession PI358934 did not exert any beneficial effect on glycemia. Indeed, mice receiving the HFD + MG_13 diet displayed a higher liver glycogen deposition than the HFD and HFD + PI358934 fed mice. Accordingly, rats fed a diet enriched with chickpea were characterized by having an increased level of glucokinase activity that correlated to a major liver glycogen deposition as compared with the HFD fed rats. These findings proposed that the consumption of chickpea could ameliorate the abnormal lipid profile [20]. Moreover, the serum level of the AST, which is a known biomarker of liver injury, was significantly higher in the HFD and HFD + PI358934 groups. An evaluation of the intrahepatic fat infiltration revealed that the lipid area in the liver tissue was significantly higher in the HFD and HFD + PI358934 fed mice as compared with the CTR mice. In contrast, previous reports have demonstrated that dietary chickpeas ameliorated the lipid profile in rats fed an HFD diet containing lard at 20% [9]. Whereas, in the present study, the high-fat diet included a higher fat level. However, similar to the in vitro data, the liver ROS content induced by the HFD diet was significantly reduced in the mice receiving the chickpea supplemented diets even though the phosphorylation level of NF-kB was reduced only in the liver of the HFD + MG_13 fed mice as compared with the HFD fed animals. Indeed, the activity of NF-kB in the liver of the HFD + PI358934 fed mice was similar to that measured in the HFD mice. Together, these findings indicate that the MG_13 accession exerted a stronger beneficial effect as compared with the PI358934 accession that contained a higher level of linoleic acid than the MG_13 accession [4]. It has been proposed that a high intake of linoleic acid can activate inflammation pathways through the accumulation of arachidonic acid and the release of several oxidative and inflammatory molecules that can play an important role in the onset of chronic diseases [42,43,44]. Nevertheless, the differential level of linoleic acid in the chickpea accessions partially explain the present data. Overall, it is plausible to hypothesize that other composition factors that differentiate these accessions are important for activating specific antioxidant and anti-inflammatory pathways that bring health benefits.

## 5. Conclusions

In conclusion, this study compares, for the first time, the effects of two different chickpea accessions revealing that only the MG_13 has beneficial responses to significantly attenuate the hepatic steatosis induced by a high-fat diet. The obtained results indicated that the beneficial actions of the single chickpea accession could be related to the presence of specific bioactive compounds or the combination of selective molecules. Hence, the identification of accession-specific phytocompounds should be an important step to explain the difference between the accessions MG_13 and PI358934. Overall, these findings highlight the importance of the genotypic, phenotypic, and nutraceutical characterization of chickpea cultivars of global food interest.

## Figures and Tables

**Table 1:** Body weight, food intake, and food efficiency ratio of CTR and high-fat diet (HFD) mice.

Parameters	CTR (12)	HFD (12)	HFD + MG_13 (12)	HFD + PI358934 (9)
**Initial body weight (g)**	22.78 ± 1.30	23.63 ± 1.16	22.68 ± 1.02	24.19 ± 0.69 *
**Final body weight (g)**	31.04 ± 1.04 ^####^	40.1 ± 3.00 ****	43.69 ± 6.08 ****	49.91 ± 4.04 **** ^####^
**Weight gain (g/d)**	0.069 ± 0.009 ^####^	0.1384 ± 0.019 ****	0.176 ± 0.036 **** ^##^	0.216 ± 0.024 **** ^####^
**Food intake (g/d)**	3.176 ± 0.286	3.036 ± 0.115	3.787 ± 0.698 **** ^####^	2.911 ± 0.226
**FER (%)**	2.185 ± 0.365 ^####^	4.557 ± 0.667 ****	4.661 ± 1.293 ****	7.423 ± 1.025 **** ^####^

Values represent the mean ± S.D., the number of mice is indicated in the brackets. * Significantly different versus CTR (**** *p* < 0.0001; * *p* < 0.05). # Significantly different versus HFD (^####^ *p* < 0.0001 and ^##^ *p* < 0.01). Food efficiency ratio (FER)

**Table 2:** Biochemical analysis of serum parameters of CTR and HFD mice.

Parameters	CTR (12)	HFD (12)	HFD + MG_13 (12)	HFD + PI358934 (9)
**Glycemia** **(mg/dL)**	87.42 ± 21.46 ^####^	214.6 ± 32.84 ****	184.6 ± 12.89 **** ^#^	209.4 ± 41.92 ****
**Insulin (μlU/mL)**	15.75 ± 4.55	26.51 ± 8.82	23.68 ± 13.41	16.05 ± 2.32
**Total cholesterol (mg/dL)**	63.00 ± 27.40	107.0 ± 9.64	186.3 ± 58.07 **	221.0 ± 27.87 ** ^#^
**Triglycerides** **(mg/dL)**	76.00 ± 39.40	128.0 ± 22.72	165.3 ± 11.37 *	181.7 ± 56.36 *
**Alkaline phosphatases (U/I)**	66.00 ± 10.54	65.33 ± 28.31	48.00 ± 15.13	64.00 ± 7.00
**AST (U/I)**	279.0 ± 130.9 ^#^	582.7 ± 130.6 *	486.3 ± 89.39	703.7 ± 49.92 **

Values represent the mean ± S.D. The number of mice is indicated in the brackets. * significantly different versus CTR (**** *p* < 0.0001; ** *p* < 0.01 and * *p* < 0.05). ^#^ significantly different versus HFD (^####^ *p* < 0.0001 and ^#^ *p* < 0.05). Aspartate aminotransferase (AST).
